# Development of a cost-effective serodiagnosis for African swine fever using solubility-enhanced recombinant p54, p30, and p72

**DOI:** 10.14202/vetworld.2025.2511-2519

**Published:** 2025-08-30

**Authors:** Simson Tarigan, Sumarningsih Sumarningsih, Atik Ratnawati, Muharam Saepulloh, Wasito Wasito, Indrawati Sendow, Harimurti Nuradji, Ni Luh Putu Indi Dharmayanti

**Affiliations:** Research Center for Veterinary Science, National Research and Innovation Agency, Jl. Raya Bogor Km. 46 Cibinong, Bogor, 16911, West Java, Indonesia

**Keywords:** African swine fever, enzyme-linked immunosorbent assay, recombinant p30, recombinant p54, recombinant p72, serodiagnosis

## Abstract

**Background and Aim:**

The rapid spread of African swine fever (ASF) in Indonesia and other Asian countries has devastated domestic and wild pig populations. In the absence of a viable vaccine, ASF control depends on strict biosecurity measures and the prompt culling of infected animals. Accurate and timely detection is therefore essential to limit disease transmission, highlighting the urgent need for reliable diagnostic tools. This study aimed to develop serological assays for ASF virus (ASFV) antibody detection using recombinant ASFV proteins.

**Materials and Methods:**

Three key ASFV structural proteins–p30, p54, and p72–were heterologously expressed in *Escherichia coli*. Protein solubility, particularly for p54, was enhanced by targeted deletion of hydrophobic domains. Recombinant proteins were purified using nickel-nitrilotriacetic acid affinity chromatography and assessed for diagnostic performance through enzyme-linked immunosorbent assay (ELISA) and immunoblotting using 114 field serum samples.

**Results:**

The solubility-optimized p54 antigen was successfully used to develop an indirect ELISA, while the insoluble p30 retained sufficient antigenicity for immunoblot-based detection. The p54-based ELISA showed high diagnostic performance, achieving an area under the curve of 0.936, with 91% sensitivity and 85% specificity. Agreement with a commercial ELISA kit was substantial (Cohen’s kappa = 0.635). Immunoblotting confirmed that all recombinant proteins maintained strong antigenicity and diagnostic specificity.

**Conclusion:**

Recombinant ASFV proteins p54 and p30 demonstrated strong potential for serological diagnostics when expressed in *E. coli*. Notably, this is the first study to report a successful domain truncation strategy for enhancing p54 solubility in *E. coli*, enabling the development of affordable, locally produced ELISA kits. The p30-based immunoblot assay serves as a confirmatory tool to strengthen ASF detection and outbreak response in resource-limited settings.

## INTRODUCTION

African swine fever (ASF) is a highly contagious and economically devastating disease that affects both domestic and wild pigs. It is caused by a large double-stranded DNA virus belonging to the *Asfarviridae* family. Among the 24 recognized ASF virus (ASFV) genotypes, genotypes I and II are the most epidemiologically significant, with genotype II responsible for the current global epizootic [1, 2]. Since its emergence in Southeast Asia in 2019, ASF has rapidly spread across the region, causing severe losses in domestic pig populations [3]. Indonesia reported its first confirmed ASF case on November 27, 2019. Molecular characterization identified the strain as genotype II, closely related to isolates from Vietnam, China, and Russia [4, 5]. As of April 2025, outbreaks have been reported in 32 of Indonesia’s 38 provinces, with major impacts in areas of intensive pig farming [6].

Due to the absence of an effective vaccine, ASF control relies primarily on stringent biosecurity measures and the culling of infected animals. Serological testing plays a crucial role in surveillance, as ASFV-specific antibodies typically appear 7–10 days post-infection and persist for extended periods, making them reliable markers of exposure [7]. Enzyme-linked immunosorbent assay (ELISA) is widely adopted for ASF antibody detection owing to its cost-effectiveness and scalability. However, conventional ELISAs often rely on ASFV-infected cell lysates, which require biosafety level-3 facilities–resources that are frequently unavailable in endemic settings [2, 8]. Confirmatory methods such as immunoblotting and immunofluorescence are labor-intensive and unsuitable for routine or large-scale application [9, 10].

Recombinant DNA technology provides a safer and scalable alternative by enabling the production of highly specific ASFV antigens, particularly p30, p54, and p72–three well-established immunogenic targets frequently used in serodiagnostics [11]. While eukaryotic expression systems yield high-quality antigens, they are often costly and technically demanding [12, 13]. In contrast, *Escherichia coli* offers a more accessible and cost-efficient platform for producing recombinant proteins. However, ASFV proteins expressed in *E. coli* often exhibit poor solubility due to hydrophobic domains and improper folding [14, 15]. Despite these limitations, several commercial ELISA kits based on different ASFV antigens are in global use, including the INgezim PPA COMPAC K3 blocking ELISA (Ingenasa/Eurofins Technologies, Madrid, Spain; targeting p72), ID Screen ASF Indirect ELISA (Innovative Diagnostics – IDvet, Grabels, France; targeting p32, pp62, and p72), and Svanovir ASFV-AB ELISA (Svanova Biotech, Boehringer Ingelheim, Uppsala, Sweden; targeting p30) [16]. In parallel, vaccine development has progressed, with Vietnam recently granting conditional approval for AVAC ASF LIVE (AVAC Vietnam Joint Stock Company, Hung Yên, Vietnam), a live attenuated vaccine showing promise for future control strategies [17].

Countries newly affected by ASF, such as Indo-nesia, often rely on imported commercial ELISA kits for diagnostic and surveillance purposes. However, these kits are expensive and vulnerable to supply chain disruptions, limiting their availability during critical outbreak periods. Moreover, while recombinant protein-based assays offer a safer and scalable alternative, their implementation in low-resource settings has been hampered by solubility challenges–especially for membrane-associated ASFV proteins, such as p54 and p72–when expressed in *E. coli*. To date, no studies have systematically applied or validated domain truncation as a strategy to enhance the solubility of ASFV antigens, particularly p54, for serological assay development in bacterial systems.

This study aimed to develop a sustainable and affordable serological platform for ASF diagnosis using recombinant ASFV proteins p54, p30, and p72 expressed in *E. coli*. To overcome solubility limitations, hydrophobic domains in p54 and p72 were strategically deleted, with a focus on enhancing the performance of serological assays. Specifically, we report the first application of a domain truncation strategy to improve the solubility of p54 in *E. coli*, enabling its use in an in-house ELISA. By combining p54-based ELISA with p30-based immunoblotting as a confirmatory tool, this platform supports the local production of diagnostic reagents and strengthens national ASF surveillance and outbreak response efforts.

## Materials and Methods

### Ethical approval

All procedures involving animals were reviewed and approved by the Animal Care and Use Committee of Balai Besar Penelitian Veteriner (Indonesian Research Institute for Veterinary Sciences [BBLITVET]) under approval number 1290/OT.050/H.5.1/09/2020, dated September 01, 2020. Serum samples were obtained as part of routine diagnostic surveillance activities. Blood collection was performed by trained personnel following standard protocols designed to minimize animal discomfort. No animals were euthanized or subjected to additional procedures for this study.

### Study period and location

The study was conducted from February to December 2020 at BBLITVET, Bogor, Indonesia.

### Gene design and plasmid construction

**Figure 1:**
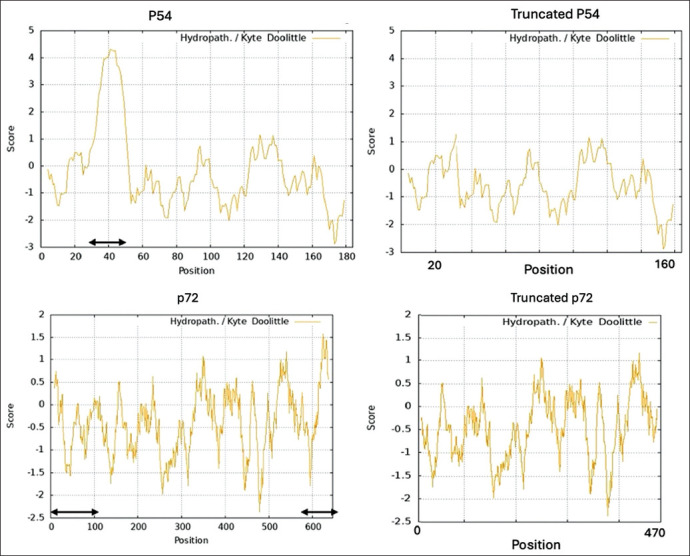
Hydropathy plots of the full-length and truncated African swine fever virus p54 and p72 proteins, generated using the Kyte–Doolittle scale. The black double-headed arrows in the full-length protein plots indicate the deleted regions in the truncated constructs. For p54 (top panels), the deleted segment corresponds to a highly hydrophobic domain, whose removal substantially improved *Escherichia coli* protein solubility. In contrast, the deleted regions in p72 (bottom panels) were not strongly hydrophobic, and their removal did not improve solubility.

The sequences used for designing the plasmids encoding p30, p54, and p72 were derived from ASFV genotype II strains, which are responsible for current outbreaks in Indonesia. The p30 sequence was obtained from a previously characterized isolate [18]. For p54 (GenBank Accession No. UTS69665.1), a hydrophobic region spanning amino acids 34–49 was removed to enhance solubility in *E. coli*. For p72, the selected fragment spanned amino acids 105–575 based on GenBank entry QGJ83444.1. Truncation regions were determined via hydropathy analysis using the Kyte–Doolittle scale with ExPASy ProtScale (https://web.expasy.org/protscale/), which identified strongly hydrophobic domains likely to impair solubility in *E. coli*. Hydropathy plots of full-length and truncated sequences are presented in Figure 1 to illustrate the deleted regions.

Codon-optimized synthetic genes for all three proteins were designed using proprietary software and reverse-translated for optimized *E. coli* expression (Genscript USA Inc., USA). Each gene was cloned into the pET30a(+) vector, which enables high-level expression through a T7 promoter and includes a 6xHis tag to facilitate purification. *E. coli* BL21 (DE3) was selected as the expression host for its compatibility with T7 systems, low protease activity, and high protein yield [19].

### Bacterial transformation and protein expression

The recombinant pET30a(+) plasmids were introduced into *E. coli* BL21 (DE3) (Sigma-Aldrich, St. Louis, MO, USA), prepared using a transformation solution containing polyethylene glycol, dimethyl sulfoxide, and 50 mM Mg²^+^, following the protocol by Chung *et al*. [20]. Unless otherwise stated, all reagents were obtained from Sigma-Aldrich.

Transformants were selected on LB agar containing 30 μg/mL kanamycin and incubated at 37°C overnight. For each construct, five colonies were randomly chosen and cultured in 5 mL LB-kanamycin broth (30 μg/mL) at 37°C with shaking at 200 rpm overnight. A 1:100 dilution of the overnight culture was inoculated into fresh LB-kanamycin broth and grown until the optical density at 600 nm (OD_600_) reached 0.4. Protein expression was induced with 1 mM isopropyl β-D-1-thiogalactopyranoside (IPTG), and incubation continued for 2 h. Cells were harvested by centrifugation at 12,000× *g* for 5 min at 25°C. Expression levels were assessed through sodium dodecyl sulfate–polyacrylamide gel electrophoresis (SDS-PAGE) by comparing IPTG-induced and uninduced cultures. The colony showing the highest recombinant protein yield was used for scale-up.

### Protein purification strategies

Cells were lysed in 50 mM sodium phosphate buffer (pH = 8.0) containing 0.5 M NaCl, and lysates were clarified by centrifugation for native purification. Soluble fractions were applied to nickel-nitrilotriacetic acid (Ni-NTA) agarose columns (Thermo Fisher Scientific, Waltham, MA, USA), washed with binding buffer, and eluted with 0.5 M imidazole. For denaturing purification, cell pellets were solubilized in 6 M guanidine hydrochloride, and proteins were purified using Ni-NTA affinity chromatography with elution in 8 M urea at pH 4.0. A hybrid approach combining guanidine solubilization and native buffer elution was also evaluated. Protein yield and purity were assessed through SDS-PAGE using 12% resolving and 4% stacking gels in Tris-glycine buffer (pH = 8.3).

### Development and optimization of ELISA

An indirect ELISA was developed based on a previously described protocol by Tarigan *et al*. [21], with modifications to optimize coating conditions for recombinant p54. Ninety-six-well Nunc Maxisorp plates (Sigma-Aldrich, Cat. No. M9410, USA) were coated overnight at 4°C with 100 μL/well of p54 (20 μg/mL) in 0.1 M carbonate-bicarbonate buffer (pH = 9.6). Protein concentration was determined by absorbance at 280 nm, with an extinction coefficient of 0.866mg^-^¹mLcm^-^¹ calculated using ExPASy ProtParam (https://web.expasy.org/protparam/).

Plates were washed thrice with phosphate- buffered saline (PBS) (pH = 7.4) containing 0.05% PBS with Tween-20 (PBST), then blocked with 150 μL of 0.1% bovine serum albumin in PBST for 2 h at room temperature (25°C). Serum samples were diluted 1:100 in a complex ELISA buffer (0.7 M NaCl, 0.05 M ethylenediaminetetraacetic acid, 3% Triton X-100, 3% Tween-20, 2% non-fat milk, 5% goat serum, and 0.1 M Tris-HCl, pH 7.4), and 100 μL was added per well for 2 h incubation at 25°C. Plates were washed four times with stringent buffer (640 μM NaCl, 3 μM KCl, 8 μM Na_2_HPO_2_ 1.5 μM KH_2_PO_2_ and 5% Tween-20).

Bound antibodies were detected using HRP-conjugated anti-pig immunoglobulin G (IgG) (1:10,000 in complex buffer), incubated for 1 h at 25°C. Color development was achieved using 100 μL/well of 2,2′-azino-bis(3-ethylbenzothiazoline-6-sulfonic acid (ABTS) substrate in phosphate-citrate buffer, pH 4.2, with 0.6% H_2_O_2_). Absorbance was measured at 420 nm using a Multiskan FC reader (Thermo Fisher Scientific, Waltham, MA, USA).

Checkerboard titrations were performed to determine optimal antigen and serum dilutions. All samples were tested in duplicate, with positive and negative control sera included on each plate. Positive control serum came from a polymerase chain reaction-confirmed ASF-infected pig, and negative serum from a non-infected animal.

### Western blotting

Western blotting was used to assess the antigenicity and specificity of the recombinant p30, p54, and p72 proteins. Proteins were transferred to nitrocellulose membranes using Tris-glycine buffer (25 mM Tris, 192 mM glycine, 20% methanol, pH 8.3) at 75 V for 2 h at 4°C. Membranes were blocked with 5% non-fat dry milk in PBST for 2 h at 25°C and incubated with sera from ASFV-infected pigs (1:100 dilution in PBST) for 2 h. After washing three times, membranes were probed with HRP-conjugated anti-pig IgG (1:5,000 in PBST) for another 2 h.

Detection was carried out using 3,3′-diaminobenzidine in Tris-buffered saline (50 mM Tris, 150 mM NaCl, pH 7.4) containing 0.01% hydrogen peroxide. Membranes were rinsed with distilled water to terminate the reaction.

### Serum sample collection

A total of 114 serum samples were analyzed, comprising 33 pre-outbreak sera and 81 samples from ASF-affected areas. These included 55 from North Sumatra and 26 from East Nusa Tenggara. All samples were obtained during routine surveillance by BBLITVET and regional partner laboratories. No formal sample size calculation was performed, as the study was exploratory in nature. ASF prevalence in the tested population was unknown due to the diverse and unlinked sources of the sera.

### Statistical analysis

The diagnostic performance of the in-house ELISA was assessed against the ID Screen ASF Indirect ELISA (Innovative Diagnostics, France), serving as the reference standard. Agreement between assays was measured using Cohen’s Kappa coefficient [22]. Receiver operating characteristic (ROC) curve analysis was conducted to calculate the area under the curve (AUC) and identify optimal OD cutoff values. Sensitivity (true positive rate) and specificity (true negative rate) were calculated at multiple thresholds. All analyses were performed using IBM SPSS Statistics version 25.0 (IBM Corp., Armonk, NY, USA).

## RESULTS

### Expression of recombinant proteins

**Figure 2:**
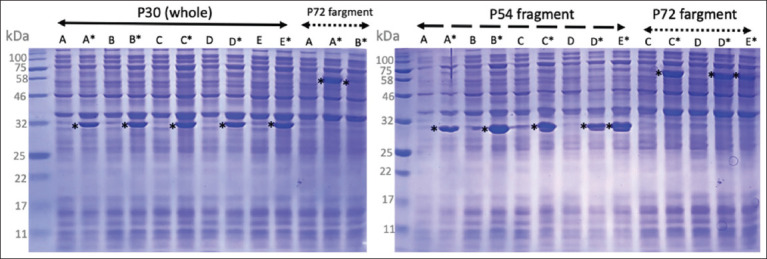
Expression of recombinant p30, p54, and p72 proteins in *Escherichia coli* cells. Five colonies (A, B, C, D, and E) from each transformation were analyzed before and after the induction of isopropyl β-D-1-thiogalactopyranoside. Samples labeled with an asterisk (A*, B*, C*, D*, and E*) represent post-induction cultures. The induced bands marked with an asterisk (*) correspond to the expected molecular weights of recombinant p30, p54, and p72.

The ASFV structural proteins p54, p30, and p72 were successfully expressed in *E. coli* BL21 (DE3). All five tested clones carrying each recombinant plasmid produced the corresponding protein upon induction with IPTG. Protein expression was minimal before induction but became clearly detectable approximately 2 h after IPTG treatment (Figure 2). The observed molecular weights of the expressed proteins exceeded theoretical predictions, likely due to fusion tags or atypical migration in SDS-PAGE. Specifically, p30 appeared at ~32 kDa (theoretical: 24.4 kDa), p54 at ~30 kDa (theoretical: 19.1 kDa), and p72 at ~58 kDa (theoretical: 54.9 kDa).

### Purification and solubility profiles

**Figure 3:**
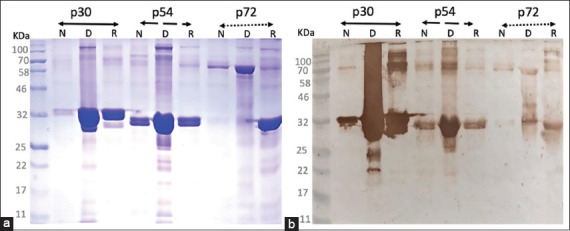
(a) Sodium dodecyl sulfate–polyacrylamide gel electrophoresis analysis of purified recombinant p30, p54, and p72 proteins using three different protocols: (1) Method N (native): Cell lysate was prepared in phosphate buffer and eluted from the nickel-nitrilotriacetic acid column using 0.5 M imidazole in phosphate buffer. (2) Method D (denaturing): The lysate was prepared in 6 M guanidine and eluted with 8 M urea (pH 4.0). (3) Method R (refolding): The lysate was prepared in 6 M guanidine and eluted with 0.5 M imidazole in phosphate buffer. (b) Immunoblot analysis of the same recombinant proteins using serum from African swine fever virus-infected pigs (1:200 dilution).

Three Ni-NTA affinity purification strategies, native, denaturing, and hybrid, were evaluated to compare yield and solubility of the recombinant proteins. Among these, the denaturing method using guanidine hydrochloride and urea elution yielded the highest protein concentrations (Figure 3a). Notably, solubility varied significantly among the three antigens. Recombinant p72 was largely insoluble, with minimal recovery under both native and hybrid conditions, confirming its limited solubility in *E. coli*. In contrast, p30 exhibited moderate solubility, with improved recovery following chaotropic extraction and subsequent native elution. Among all constructs, p54 demonstrated the highest solubility, which was significantly enhanced by targeted deletion of its hydrophobic domain.

### Immunoblotting analysis

**Figure 4:**
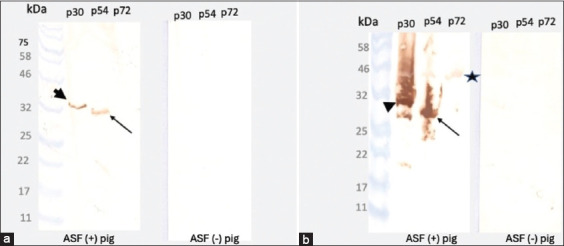
Immunoblot analysis of recombinant p30, p54, and p72 proteins probed with serum from African swine fever virus (ASFV)-infected and ASFV-negative pigs. (a) Serum diluted 1:800. (b) Serum diluted 1:100. Positive reactivity was observed only in ASFV-infected sera, demonstrating antigen specificity. Notably, reactivity against p72 was clearly observed only at the 1:100 dilution of ASFV-infected serum (indicated by a star).

Immunoblot analysis verified that all three recombinant proteins, p30, p54, and p72, retained strong antigenicity and specificity. Sera from ASFV-infected pigs consistently recognized all three proteins across different purification protocols. No cross-reactivity was observed when sera from ASFV-negative pigs were used, even at the highest serum concentrations or lowest dilutions (Figures 3b and 4). These results confirm that the recombinant antigens preserved diagnostically relevant epitopes capable of differentiating infected from uninfected animals.

### ELISA performance evaluation

Given its high solubility and yield, p54 was selected for the development of the in-house ELISA. The p54-based indirect ELISA demonstrated strong diagnostic performance, effectively distinguishing ASFV-exposed pigs from seronegative controls. ROC analysis yielded an AUC of 0.936 (95% confidence interval: 0.883–0.989; standard error [SE] = 0.027), indicating excellent overall accuracy (Table 1). Comparison with the commercial ID Screen ASF Indirect ELISA revealed substantial agreement, with a Cohen’s kappa value of 0.635 (SE = 0.081; T = 7.053; p < 0.001) (Table 2). At the optimal OD cutoff of ≥0.790, the in-house assay achieved 91% sensitivity and 85% specificity (Table 3). Using a more permissive cutoff (0.409) increased sensitivity to 100% but reduced specificity to 57%. Conversely, a stricter cutoff (0.987) improved specificity to 90% but decreased sensitivity to 78%. These results underscore the diagnostic utility of the p54-based ELISA as an affordable and scalable tool for ASF serological surveillance, particularly in resource-limited settings.

**Table 1:** AUC demonstrating the discriminating power of the p54-based ELISA.

AUC	Standard error	Asymptotic signal (H0: true area = 0.5)	95% CI lower	95% CI upper
0.936	0.027	0.000	0.883	0.989

AUC = Area under the curve, ELISA = Enzyme-linked immunosorbent assay, CI = Confidence interval

**Table 2:** Measurement of agreement between p54-based ELISA and ID Screen ASF indirect ELISA (Innovative Diagnostics, France).

Kappa value	Asymptotic standardized error^a^	Approximate T^b^	Approximate significance
0.635	0.081	7.053	0.000

a The null hypothesis is not assumed (p < 0.001).

b Using the asymptotic standard error and assuming the null hypothesis (p < 0.001). ASF = African swine fever, ELISA = Enzyme-linked immunosorbent assay

**Table 3:** Sensitivity and specificity of the p54-based ELISA at different OD cut-off values.

Cutoff (OD≥)	Sensitivity (%)	Specificity (%)
0.409	100	57
0.790	91	85
0.987	78	90

ELISA = Enzyme-linked immunosorbent assay, OD = Optical density

## DISCUSSION

### Diagnostic potential of recombinant antigens

Rapid and accurate diagnosis is critical for controlling ASF, particularly in regions facing emerging or ongoing outbreaks [23]. In this study, ASFV structural proteins p54, p30, and p72 were successfully expressed in *E. coli*, and their diagnostic utility was assessed using both ELISA and immunoblotting platforms. The p54-based ELISA demonstrated high diagnostic accuracy, with an AUC of 0.936, indicating a strong ability to differentiate between positive and negative sera. The incorporation of a p30-based immunoblot as a confirmatory tool further enhanced diagnostic reliability. This two-tiered approach offers a practical framework for improving ASF serological surveillance.

### Comparative performance and assay variability

Although the AUC was high, the sensitivity and specificity values did not reach ideal thresholds. These findings align with previous studies by Fernandez Pacheco *et al*. [24], such as one reporting a 72.7% sensitivity for a p54-based ELISA. Variations in antigen preparation, protein folding, and assay conditions may contribute to the observed differences in diagnostic performance. In contrast, another study by Gallardo *et al*. [25] using insoluble p54 expressed in *E. coli* achieved significantly higher metrics, 98% sensitivity, 97% specificity, and a Kappa value of 0.95, –highlighting the importance of expression strategy and assay refinement.

### Protein-specific characteristics and diagnostic utility

#### p30: Immunoreactive but poorly soluble in E. coli

The p30 protein is a well-known immunogen that localizes within the virion and extracellular environment during ASFV infection. Its expression in eukaryotic systems such as *Trichoplusia ni* larvae has yielded high-quality ELISA antigens [12, 25]. Consistent with prior findings, p30 remained largely insoluble when expressed in *E. coli* in this study. Attempts to improve solubility via hydrophobic region deletion were unsuccessful by Giménez-Lirola *et al*. [14]. Nevertheless, the recombinant p30 retained strong immunoreactivity in immunoblot assays, reaffirming its value as a confirmatory antigen [15, 26].

#### p54: Solubility enhanced by domain truncation

Although p54 is classified as a structural protein, it functions as an inner envelope transmembrane protein with a molecular weight of 25 kDa [27]. Due to its membrane-associated nature, p54 typically exhibits low solubility when expressed in prokaryotic systems [25, 28]. In this study, solubility was significantly improved by strategically deleting a key hydrophobic domain. To the best of our knowledge, this is the first report of a domain truncation strategy successfully enhancing p54 solubility in *E. coli*. These results demonstrate the feasibility of rational protein engineering to improve membrane protein expression in bacterial systems for diagnostic purposes.

#### p72: Persistent insolubility in bacterial systems

As the most abundant ASFV structural protein, p72 accounts for over 30% of the virion mass and is frequently targeted for both diagnostics and vaccine development [29]. While soluble p72 has been produced in mammalian expression systems such as HEK293F cells, the protein remains insoluble in *E. coli*, even after extensive truncations at both the N- and C-termini [30]. This study’s results were consistent with those observations, p72 constructs remained largely insoluble despite domain deletions, further underscoring the challenge of using p72 in *E. coli*-based diagnostic platforms.

### Study limitations and implications for future work

One major limitation of this study was the relatively small sample size, primarily due to logistical constraints in obtaining well-characterized ASFV-positive field sera. The sample size was not statistically calculated, and the true prevalence of ASF among the tested population was unknown. This limitation may have contributed to the modest sensitivity, specificity, and Cohen’s kappa values, as small datasets are more prone to variability and classification errors [22].

Despite this limitation, the ELISA developed in this study shows strong potential. Future assay optimization, particularly in terms of antigen coating conditions, buffer composition, and incubation protocols, may further enhance diagnostic performance. Although this study focused on domain deletion to improve p54 solubility, additional strategies such as fusion tags, mild detergents, or co-expression with chaperone proteins should be explored to improve solubility and yield across all antigens [31–33]. These refinements could facilitate broader application of this diagnostic platform, especially in resource-limited settings where rapid, scalable, and affordable ASF detection is urgently needed.

## CONCLUSION

This study successfully developed a cost-effective serological diagnostic platform for ASF using solubility-enhanced recombinant ASFV proteins expressed in *E. coli*. Among the three antigens tested, the domain-truncated p54 protein demonstrated the highest solubility and was effectively used to develop an in-house ELISA. This assay achieved strong diagnostic performance, with an AUC of 0.936, sensitivity of 91%, and specificity of 85%, and showed substantial agreement with a commercial ELISA kit (Cohen’s kappa = 0.635). The p30 protein, although largely insoluble, retained robust antigenicity and proved useful in confirmatory immunoblotting. In contrast, p72 remained consistently insoluble despite truncation, limiting its utility in *E. coli*-based platforms.

The findings highlight the feasibility of using *E. coli*-expressed ASFV antigens to establish low-cost, scalable diagnostics that reduce reliance on imported commercial kits. The solubility enhancement of p54 through domain truncation represents a novel and effective strategy, enabling downstream applications in surveillance and outbreak management, particularly in resource-limited settings.

A key strength of the study lies in its practical approach to local reagent production using accessible bacterial expression systems, which can be readily implemented in endemic countries. In addition, the integration of both ELISA and immunoblotting enables a tiered diagnostic framework for initial screening and confirmatory testing.

In conclusion, this platform lays the groundwork for decentralized ASF serodiagnosis and provides a valuable model for developing similar diagnostic tools for other transboundary animal diseases. Further optimization and field validation across broader populations will be critical for its implementation in routine surveillance programs.

## AUTHORS’ CONTRIBUTIONS

ST: Conceptualization, investigation, supervision, and drafted the manuscript. NLPID: Conceptualization and drafted the manuscript. SS, AR, WW, HN, and IS: Investigated and drafted the manuscript. MS: Investigation. All authors have read and approved the final manuscript.
